# Rambles in Europe in 1839, with Sketches of Prominent Surgeons, Physicians, Medical Schools, Hospitals, Literary Personages, &c.

**Published:** 1841-07

**Authors:** 


Art. XIII.—*Rambles in Europe in* 1839, *with Sketches of Prominent Surgeons, Physicians, Medical Schools, Hospitals, Literary Personages, &c.* By William Gibson, m.d., Professor of Surgery in the University of Pennsylvania, &c.—*Philadelphia*, 1841. 8vo, pp. 309.

We almost wish, for once, that we of this Journal were, in reality, what our ingenious but rather nervous friend, Dr. Marshall Hall, in the preface to his last work, calls us, "boys," in order that we might be able to give an impartial notice of Dr. Gibson's amusing little volume. But really our rambler has done us the honour to sketch among his "prominent surgeons and physicians" so many of us, that we know not where to look for a reviewer who might be supposed reasonably free from bias. Nay more, if Dr. Gibson has not tainted the very fountain-head of our critical justice with "sweet, sweet poison," he has, at least, done enough to make its award in his own case suspicious, by pronouncing of our Journal that "of all the foreign reviews it is incomparably the best, being conducted on the fairest and most liberal principles," &c. We must, therefore, content ourselves with giving such a cold and meager notice of it, as even Dr. Hall himself might almost be brought to believe impartial, simply recommending our readers to judge for themselves of its merits, after telling them that it contains graphic portraits from the life of almost every medical man of eminence in London, Paris, Dublin, Edinburgh, and the English provincial cities. We think we may safely add, without any risk of misinterpretation of our motives, that the "Rambles" are incontrovertibly the production of the best-natured of travellers. Dr. Gibson seems to be naturally of such a fortunate temperament, as to relish only what is commendable, passing over, with the happy instinct of an Epicurean, all that is either evil or disagreeable. Consequently, his pictures are almost all *couleur de rose*, without a single trace of that sable tincture which, we are ashamed to say, too many of our own countrymen have employed in delineating the men and manners of America. But this return of cordial kindness and liberality on the part of our transatlantic brethren, is, at once, the best lesson and the severest punishment they can bestow on the detractors of their own great country. It would be manifest injustice to the author of this work not to say, that it is as cleverly as it is kindly written, and that the members of the profession of his own country and ours, will find in its perusal not a little of that innocent and happy dissipation from the toils and cares of business, which seems to have predominated in its composition.

After all, we find we cannot close this odd and amusing volume without presenting our readers with a specimen of its style and manner. We dip in at random, merely taking the precaution of going as far as possible from the seat of our government for our illustrations:

I. *Lisfranc of Paris.* "He is a big, burly, narrow-shouldered man, more than six feet high, negligent in dress, awkward in gait, uncouth in manners, and loud and boisterous in discourse. In his lectures he is said to be so unsparing of his brethren, and, in hospital practice, so harsh towards his patients, as to be unpopular with both. I cannot say whether those charges are well founded, but am inclined to believe them exaggerated, inasmuch as I saw nothing, beyond the natural want of polish in the man (increased, I thought, by affectation of wishing to appear worse than he really was), from which I should have drawn such a conclusion. I was seated, with a bevy of young medical friends, beneath the boughs of a wide spreading elm, on a delightful summer morning, the 18th of June, and saw him, for the first time, as he entered the hospital gate, and sauntered slowly along the gravelled walk of the long and wide avenue, leading to the ward containing his female patients. His head, covered with a rusty black and red cap, which, in shape of a tea-cup, stuck like a plaster to the summit of his crown; his long-waisted, scanty, snuff-coloured coat, dangling about his heels, and tapering away to sharpness, like the tail of a kite; his curiously contrived pantaloons, loose and bagging about his hips, and at each stride fluttering to the wind; his long, shovel-shaped shoes, scattering pebbles as he walked from right to left; his arms standing out from his body, like the handle of a pump, conjoined with his outstretched flexible neck, which swung to and fro beneath the pressure of his lengthy and wedge-shaped visage, presented one of the most ludicrous spectacles I ever beheld. He cast an enquiring sidelong glance at our group as he passed, which seemed to indicate, I thought, a belief that we were amusing ourselves at his expense, for he instantly bristled up, and with averted head hurried out of sight. . . . . He called the roll to ascertain that all his *internes* or house pupils were mustered at their posts, and refused to proceed until a delinquent, who was in bed, taking a morning nap, was brought to the scene of action. He then began a clinical discourse, explaining the general nature of the diseases before him, waxing warmer and warmer as he proceeded, and gradually raising his stentorian voice until its tones seemed to shake the foundations of the old building and startle the very rafters above our heads; whilst he, peering and scanning from right to left the looks of his auditors, with great self-satisfaction, seemed to enquire into the effect his sesquipedalian words and thundering sentences may have produced upon their minds." (p. 79.)

II. *Dr. Graves of Dublin.* "I quickly perceived a certain indescribable something in his keen penetrating eye, arrangement of the muscles of the face, and mobility of the tip of his long and delicately-formed aquiline nose, plainly indicative of humour, high spirits, and quizzical propensities, with ample power to subdue or bring them forward at pleasure. And I was not deceived; for of the many little stories I picked up in the land of blunders and bulls, some of his were the best; told so oddly and quaintly, with the true Tipperary accent on his tongue, when he chose to place it there, that I can't to this day help laughing when I think of the queer questions put to some of his hospital patients and the funny dialogues carried on between them, such as 'Patrick, my dear, and how are you to-day? Oh bravely, yer honour, and how's yer honour's self? Did the pills throuble you last night, my dear? Faix, and they handled me nately. Where is your tongue, my dear? In my mouth, yer honour. And are you maning to bring it out? Troth and I am. Can you breathe, my lad? Av coorse, yer honour, and sure you might see that I can. That's right, my dear, and there's a pinch of snuff for you, Pat. Thank you kindly, and long life to yer honour; troth and yer a rale jantleman of a doctor.' And so he proceeded from bed to bed, and no language can convey the odd mixture of kindness, and humour, and familiarity, and good sense, and soberness, and dexterity with which he managed to draw from the patients the history of their disease, the symptoms, &c. All this and a great deal more was enacted in the Meath Hospital, where I spent a forenoon with him, after partaking, at his house, of a plentiful and most luxurious breakfast, such as would have done honour to the best Virginia housewife, or to the table of Mrs. Randolph herself. After this I saw as much of Dr. Graves as a fortnight in Dublin would allow. That is, I saw him every day, either at his own house, or at my lodgings, or the Meath Hospital, where he may be found, every morning, peeping and prying into every hole and corner of the building, cracking jokes with the patient or pupils, or old women, or poring over intently some medical production, or volume of natural history, or book of travels; for he is very fond of such studies, and took great delight in asking all sorts of questions about our Indians, and lakes, and trees, and prairies, and cataracts, and great rivers, and buffaloes. And, from all I did see of him, I felt justified, I thought, in jumping to the conclusion that he is a man of very extraordinary abilities, sufficient to enable him to master any subject to which he may devote his attention, and to sift thoroughly any medical case and follow out successfully its treatment; but that he is seldom capable for any length of time of devoting himself, soul and body, to any given point or subject; that he is too fond of analogy and of drawing conclusions from solitary facts, so that his listener is always left in doubt as to the certainty of his deductions, however striking and brilliant they may be, as they generally are, from wanting full confidence in his premises." (pp. 15-6.)

